# Comparative Analysis of Interfacial Adaptation and Depth Penetration of Recent HiFlow versus Regular Bioceramic Sealers in Conjunction with BC Gutta-Percha Points Using Two Different Obturation Techniques—A Preliminary Report of an Ex Vivo Study

**DOI:** 10.3390/jfb15050134

**Published:** 2024-05-17

**Authors:** Sawsan T. Abu-Zeid, Ruaa A. Alamoudi

**Affiliations:** 1 Endodontic Department, Faculty of Dentistry, King Abdulaziz University, Jeddah 22252, Saudi Arabia; ralamoudi1@kau.edu.sa; 2 Endodontic Department, Faculty of Dentistry, Cairo University, Giza 12345, Egypt

**Keywords:** bioceramic root canal sealers, adaptation, depth penetration, flow, film thickness

## Abstract

This study aimed to assess the adaptability and penetration depth capacity of recent bioceramic systems, including regular EndoSequence (BC) versus HiFlow (BCH) sealers in the presence of BC points. A total of 54 single-rooted teeth were instrumented and obturated with either the cold or warm compaction technique (*n* = 9), using either BC, BCH, or AH Plus (AHP) combined with BC points. The adaptation, film thickness, and gaps/voids were evaluated by scanning electron microscopy. The sealer/dentin interface was evaluated by Raman spectroscopy, and depth penetration was evaluated by a confocal laser scanning microscope. According to the normality test, the data were statistically analyzed by ANOVA or Kruskal–Wallis and Mann–Whitney U tests at *p* < 0.05. BCH sealer showed the significantly thinnest film with the greatest flow (*p* > 0.001), with further improvement when subjected to the warm compaction technique. Moreover, it exhibited close adaptation with deep penetration into radicular dentin, forming a tag-like structure. The Raman spectra also indicated close contact with the dentin surface. The use of BC sealer with BC points exhibited homogenous, single-unit obturation, either with a cold or warm technique. Furthermore, the use of the warm compaction technique with BCH sealer achieved a gap-free interface associated with tag-like structures, which exhibit the monoblock phenomenon.

## 1. Introduction

Warm vertical compaction is a technique that was designed by Schilder in 1967 to produce three-dimensional (3D) obturation within complex root canal anatomy and dense obturation compared to the lateral compaction technique [1]. Although this technique improves the homogeneity and compaction of gutta-percha obturating material into the canal space [1], it is still essential to use a sealer to compensate for the lack of gutta-percha adhesion properties and fill minute irregularities and voids within the root canal system [2,3]. Previous studies have shown that optimal obturation using a root canal sealer leads to a better prognosis [1,4].

The adequate adaptation of root canal obturation depends on the sealer’s properties, including its ability to adhere well to radicular dentin and penetrate deeply into dentinal tubules, which promotes a gap-free sealer/dentin interface [5,6]. AH Plus (AHP, Dentsply De Trey Gmbh, Konstanz, Germany), a resin-based sealer, is considered the gold standard sealer due to its physical and chemical properties [7]. However, it lacks chemical adhesion to the gutta-percha [7], and for this reason, as well as its bioactivity [8], calcium silicate sealers have gained popularity recently.

Bioceramic sealer (BC) is a tricalcium silicate-based sealer introduced to endodontics in 2009 [9,10]. EndoSequence (BC) sealer (Brasseler USA, Savannah, GA, USA) is a premixed, injectable single paste composed of tri- and di-calcium silicates, calcium phosphate monobasic, tantalum oxide, zirconium oxide, and thickening agents [6,11,12]. It possesses excellent biocompatibility, antimicrobial activity, and bioactivity [6,10,13,14], along with good dimensional stability and a lack of shrinkage after setting [15,16]. However, a previous study reported that the flowability of BC sealer reduces when the temperature rises up to 140 °C during thermal obturation, from 22.9± 0.9 to 13.3 ± 1.5 mm [17]. Thus, a new formulation of BC sealer has been developed called EndoSequence BC HiFlow (BCH) sealer, which is chemically equivalent to BC sealer but has 20% greater radiopacity and lacks a thickening agent. It is designed to exhibit lower viscosity with a high heat resistance [18,19].

Gutta-percha (GP) points can be used with BC sealer. However, the manufacturer claims that BC-coated GP points, which are impregnated and coated with BC nanoparticles, increase the bonding efficiency with BC Sealer, achieve a gap-free seal, and enable 3D bonded obturation [18]. According to manufacturer’s recommendation, BC points (Brasseler USA, Savannah, GA, USA) should be used with standard BC sealer during the cold compaction technique, to achieve 3D bonded obturation [18]. They can also be used with AH Plus. However, the manufacturer advocates using the new BC point 150 series with BCH during the warm compaction technique to avoid dryness and withstand high temperatures [18,20].

The efficacy of using BC sealer with BC-coated GP points at high temperatures is currently unclear and under investigation, despite reports exhibiting appropriate qualities under conventional test setting [21,22].

The current study aimed to compare the adaptability and penetration depth capacity of two contemporary bioceramic sealers (regular EndoSequence (BC) and HiFlow (BCH)) versus the traditional epoxy resin AH Plus (AHP) sealer (as the control group) when BC-coated GP points were present. The null hypothesis was that there was no difference between the three sealers with two obturation techniques.

## 2. Materials and Methods

### 2.1. Specimen Preparation

The procedures of this study were approved by the King Abdulaziz University ethical committee (# 246-05-21). All experiments were conducted in accordance with relevant guidelines and regulations. Patients’ informed consent was waived by the King Abdulaziz University ethical committee as the teeth were extracted for orthodontic purposes and randomly selected for this study. The samples were prepared in the Advanced Technology Dental Research Laboratory at the Faculty of Dentistry, KAU.

A total of fifty-four mandibular human mature single-rooted premolars were collected. Periapical radiographs were taken in bucco-lingual and mesio-distal directions to confirm the presence of a single root canal with a curvature less than 10 degrees [23]. The teeth were stored in deionized water with thymol solution until used. Each tooth was decoronated using a diamond disk to standardize the root length at 14 mm. The working length was established by inserting a #10 K-file (Dentsply Maillefer, Ballaigues, Switzerland) until it was visible from the apical foramen, and then 1 mm was subtracted. All root canals were instrumented using ProTaper Universal rotary instruments (Dentsply Maillefer, Ballaigues, Switzerland) up to F3 (30/09). Throughout the instrumentation procedure, irrigation with 5 mL of 5.25% sodium hypochlorite (NaOCl) (Sigma-Aldrich, St. Louis, MO, USA) was performed. A final flush with 5 mL of 17% ethylenediaminetetraacetic acid (EDTA) solution (Sigma-Aldrich, Saint Louis, MO, USA) for 1 min, followed by 1 mL of 5.25% NaOCl for 1 min, was carried out. The canals were dried with paper points (Brasseler USA, Savannah, GA, USA).

The instrumented root canals were randomly divided into two experimental groups: premixed injectable BC and BCH, and one control group; AH Plus (AHP, Dentsply De Trey Gmbh, Konstanz, Germany). Each group was further divided into two subgroups according to the obturation technique (n = 9): lateral compaction technique and warm vertical compaction technique.

The root canals were obturated in a standardized manner by the same operator (RA). For the lateral compaction technique groups, BC points (Brasseler USA, Savannah, GA, USA) were used with BC and AHP sealers, while BC points 150 series (Brasseler USA, Savannah, GA, USA) were used with BCH sealer (as recommended by the manufacturer). Approximately 0.05 mL of sealer was injected into the root canal, and the matched gutta-percha cone (30/09) was lightly coated with sealer and inserted into the canal to the full working length. The root canal was filled with fine-sized BC GP points and laterally compacted using a size 25 finger spreader (Dentsply Maillefer, Ballaigues, Switzerland) inserted 2 mm short of the working length. Compaction continued until the spreader could not be inserted more than 2 mm into the canal. BC GP points were cut at the canal orifice.

**Figure 1:**
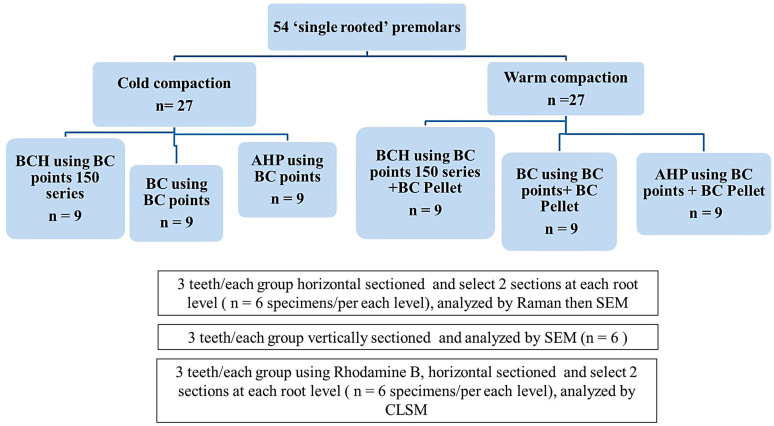
Flowchart describing the distribution of the selected teeth among the methods of evaluation.

For the warm vertical compaction technique, the selection of master GP points and sealer was similar to the lateral compaction group. However, a system B unit (SybronEndo, Orange, CA, USA) was used at 200 °C to remove the BC GP point 3 mm short of the working length, followed by vertical condensation with a Buchanan hand plugger (Sybron Endo, Orange, CA, USA). Additional sealer was applied, and back-fill was performed with the Super-Endo Beta Main Unit (B&L Biotech USA, Bala Cynwyd, PA, USA) using BC pellets (Brasseler USA, Savannah, GA, USA) at 200 °C, while withdrawing the tip up to the coronal level to achieve complete root canal obturation. Finally, vertical compaction was applied using a Buchanan hand plugger (Sybron Endo, Orange, CA, USA). Radiographs were taken in the bucco-lingial and mesio-distal aspects to assess the quality of root canal filling. The filling was considered satisfactory if it appeared dense without voids and extended within 1 mm from the root end. All specimens were kept in an incubator at 37 °C and 100% humidity for 10 days to allow the sealers to set. The obturated root canals were distributed among the evaluation methods according to the flowchart described in Figure 1.

### 2.2. Dentinal Tubules Adaptation and Penetration Test of Sealers Using Scanning Electron Microscopy (SEM)

Six obturated roots from each group were centrally and vertically embedded in orthodontic resin (Dentsply Caulk, Milford, DE, USA). Three of them (n = 3) [2] were horizontally sectioned into 2 ± 0.1 mm thick slices (two sections at each root level: apical, middle, and cervical totaling 6 slices per tooth). This was performed perpendicular to their long axis using a low-speed saw (Micracut 125 Low Speed Precision Cutter, Metkon Inst Ltd, Bursa, Turkey) with copious coolant irrigation. According to the sample size, the recommended specimen size was 6 per group to ensure sufficient significance with an alpha-type error of 0.05 and beta power of 0.80 [2,24]. The sample surfaces were smoothed with sandpaper for 10 s to reduce surface roughness. To evaluate the interface between the dentin wall and the sealer, each slice was examined under SEM (Octane pro, 7.2/15252, EDAX, Ametek Material Analysis Division, Mahwah, NJ, USA) at magnification of 500–2000×. The overall quality of the obturation was also assessed. The integrity of the sealer interfaces was evaluated concerning their homogenous interface (either at the sealer/dentin or sealer/gutta-percha interface) and/or the presence of gaps or voids. The sealer film thickness was also measured using image J software (Java-based image processing program, version 1.44, 64-bit Java 1.8.0_112, National Institute of Mental Health, Bethesda, MD). The evaluation was performed by one author (S.A) and rechecked by the second author (RA). Using the Kappa index, the reliability of the intra-examiner (ĸ = 0.92) and inter-examiner (ĸ = 0.86) was considered.

The remaining three samples were longitudinally split. Using a low-speed saw with copious coolant irrigation, a shallow groove was made in the enamel along the entire length of the buccal and lingual wall surfaces, without touching the dentin. The tooth was then split into two halves using a chisel, and examined under SEM. The lateral sides of each root segment with attached root filling were examined to evaluate the intimate contact between the sealer and dentin wall, as well as the diffusion of the sealer within the dentinal tubules, with a magnification range of 1500–2000×. The presence or absence of sealer penetration into dentinal tubules was also determined.

### 2.3. Raman Spectroscopy Analysis

The cross-section specimens obtained for SEM were initially analyzed using Raman spectroscopy (WITec GmbH, Ulm, Germany) at an excitation wavelength of 785 nm and a laser power of 25 mw. For each specimen, three spectra were recorded: one at the sealer/gutta-percha interface, another at the sealer/dentin interface, and a third at the adjacent dentin. The spectra were then analyzed to identify the interacting peaks at the sealer/dentin interface with those of sealer on one side, and those of the adjacent dentin on the other side.

### 2.4. Depth Penetration Analysis Using Confocal Laser Scanning Microscopy (CLSM):

Eighteen instrumented roots were obturated using the same techniques as described above (n = 3 per group), with the addition of approximately 0.1 mg/mL Rhodamine B dye (Loba Chemie PVL. Ltd., Mumbai, India) to the sealer [25]. Cross-sectional specimens (6 sections/tooth) were prepared following the previously described method and examined using confocal microscopy (Leica Microsystems CMS GmbH, Mannheim, Germany) at a 540 nm excitation wavelength and 590 nm emission wavelengths. The depth of sealer penetration was analyzed through image analysis using LAS-X software, version 3.7.4 for the same confocal microscopy (Leica Microsystems CMS GmbH, Mannheim, Germany). The maximum depth of penetration and the total percentage of penetration were evaluated [26].

### 2.5. Flow/Film Thickness Test

The flow of each sealer was tested according to ISO 6876/2012 [27] for dental root canal sealing materials. Five specimens were taken for each sealer. A volume of 0.05 ± 0.005 mL of premixed sealer (BC, BCH) or mixed sealer (AHP) was placed at the center of a glass plate (40 × 40 × 5 mm^3^). After three minutes, a second glass plate weighing 20 g and an additional 100 g were placed centrally on top of the sealer. This assembly was then kept at either 25 °C or 100 °C [28] in an electric heating oven (Tianjin Zhonghuan Co., Ltd., Tianjin, China). After 10 min from the start of mixing, the load was removed, and the minimum and maximum diameters of the sample discs were measured using a digital caliper (Cole-Parmer Canada Inc., Montreal, QC, Canada) with a resolution of 0.01 mm. A disc with a diameter of at least 17 mm needed to be obtained. If the discs were not uniformly circular or if the maximum and minimum diameters were not within 1 mm, the test was repeated. The thickness of the double glass slabs containing the sealer in between was measured (Ts) using the same digital caliper. The thickness of the empty double slabs without sealer was also determined (T0). The film thickness of each sealer was calculated as Ts-T0 [29].

### 2.6. Statistical Analysis

According to the normality test (Kolmogorov–Smirnov *p* > 0.05), the data of film thickness, flow, and maximum depth penetration followed the normal distribution; thus, the ANOVA and post hoc tests were used at a significant difference of *p* < 0.05. However, sealer penetration percentage did not follow the normal distribution, and nonparametric tests (Kruskal–Wallis and Mann–Whitney U) were used.

The assessment of all evaluation tests was performed with one author (S.A) and rechecked by the second author (RA). Using the Kappa index, the reliability of the intra-examiner (ĸ = 0.92) and inter-examiner (ĸ = 0.86) was determined.

## 3. Results

### 3.1. SEM

When the sealer was subjected to the cold compaction technique, BCH exhibited the significant thinnest film at all root levels (cervical, middle, and apical at *p* < 0.001) with mean values of 25.98 ± 5.58, 21.57 ± 7.14, and 21.56 ± 4.46 µm, respectively, followed by BC (40.98 ± 6.29, 33.66 ± 5.29, and 26.34 ± 3.81 µm, respectively). Whereas, AHP had the significantly thickest film (*p* < 0.001), with mean values of 65.39 ± 6.37, 64.89 ± 9.53, and 57.84 ± 9.75 µm, respectively. In addition, there was no significant difference in film thickness between the three root levels for each sealer. In contrast, when the sealers were subjected to high temperatures using the warm compaction technique, the film thickness was significantly reduced compared to the cold compaction technique (*p* < 0.001). Both BCH and AHP sealers showed the significantly thinnest film thickness at all three root levels with mean values of (22.93 ± 4.27, 15.66 ± 5.4, and 11.3 ± 1.72 µm for BCH and 22.55 ± 2.09, 18.53 ± 2.77, and 10.67 ± 0.85 µm for AHP, respectively) compared to BC sealer (24.01 ± 6.22, 21.15 ± 3.4, and 16.7 ± 1.54 µm respectively), at *p* < 0.001 (Table 1).

**Figure 2:**
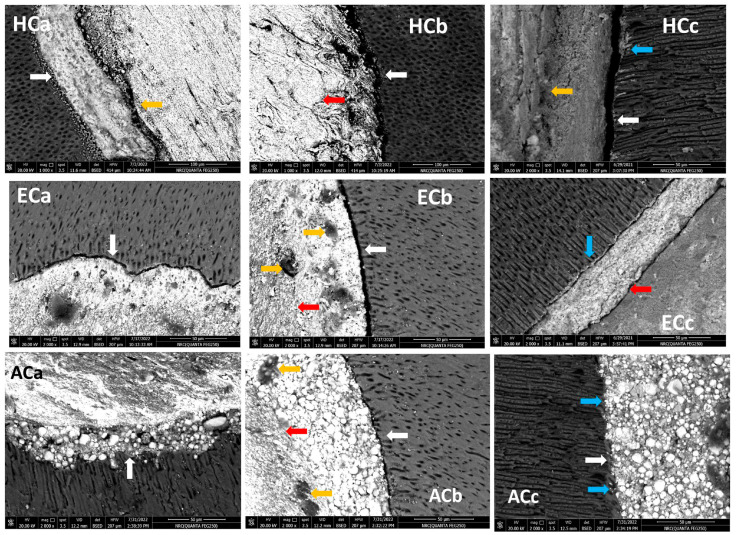
Scanning electron microscope photographs (at ×2000) of root canals obturated with BCH (H), BC (E), and AHP (A) subjected to cold compaction (C) obturation technique. Horizontal sections (a and b) and longitudinal sections (c). The white arrow points to sealer/dentin interfaces, the red arrows point to sealer/gutta-percha interfaces, the orange arrows point to the voids within sealer layer, and the blue arrows point to the extension of sealer into dentinal tubules.

Upon cold compaction, the BCH/dentin interface exhibited intimate contact (gap-free) at a few sides (white arrow in Figure 2 HCa), while small gaps at other sides ranging between 16.55 and 7.21 µm were present (white arrows in Figure 2: HCb and HCc). Meanwhile, the BCH/gutta-percha interface revealed small distributed voids in some areas (orange arrow in Figure 2 HCa and HCc). In addition, there was evidence of BCH penetration into dentinal tubules, forming tag-like structures (blue arrow in Figure 2: HCc). The BC/dentin interface revealed gaps at all sides ranging between 6.68 and 2.99 µm (white arrows in Figure 2 ECa and ECb), without evidence of tag-like structures. However, in the longitudinal section, some BC sealer was observed penetrating at a shallow depth within dentinal tubules (blue arrow in Figure 2 ECc). Meanwhile, there was no line of demarcation at certain areas of both the BCH/gutta-percha and BC/gutta-percha interfaces (red arrow in Figure 2: HCb and ECb, respectively). The BC/gutta-percha interface also showed voids within the sealer layer (orange arrows in Figure 2 ECb). The AHP/dentin interface revealed an intimate contact (gap-free) at a few sides (white arrow in Figure 2: ACa), while a minute gap was present at others ranging between 2.34 and 0.65 µm (white arrow in Figure 2 ACb). There was close contact with the gap-free contact at the AHP/gutta-percha interface, with a line of demarcation between them, and a few voids (red and orange arrows, respectively, in Figure 2 ACb). No tag-like structures were seen in the longitudinal section (blue arrows in Figure 2 ACc).

**Figure 3:**
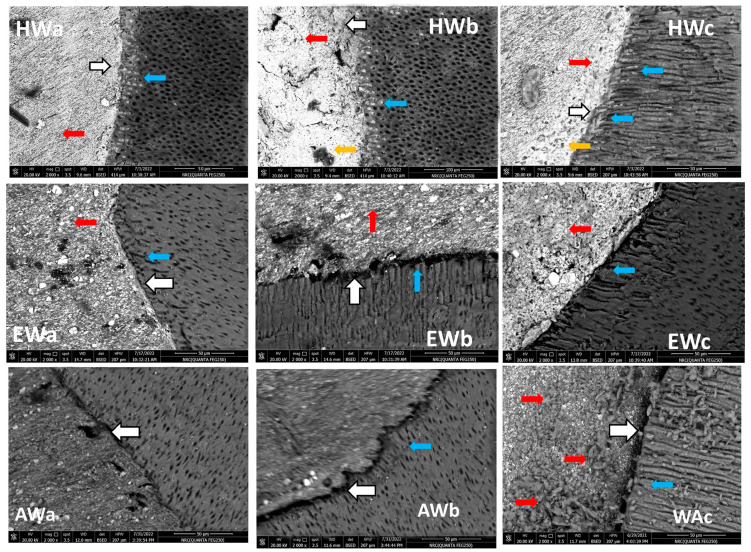
Scanning electron microscope photographs (at ×2000) of root canals obturated with BCH (H), BC (E), and AHP (A) subjected to warm compaction (C) obturation technique. Horizontal sections (a and b) and longitudinal sections (c). The white arrow points to sealer/dentin interfaces, the red arrows point to sealer/gutta-percha interfaces, the orange arrows point to the voids within sealer layer, and the blue arrows point to the extension of sealer into dentinal tubules.

In the warm vertical compaction technique, there was an intimate contact (gap-free) at the BCH/dentin interface on all sides (white arrows in Figure 3: HWa–HWc), and there was evidence of BCH penetration into the dentinal tubules forming tag-like structures (blue arrows in Figure 3: HWa–HWc). In addition, a homogenous obturation without a line of demarcation was observed between the BCH and gutta-percha core (red arrows in Figure 3 HWa–HWc), while only small voids were detected within the BCH layer (orange arrows in Figure 3: HWb and HWc). The BC/dentin interface detected either close contact or a small gap in a range between 7.08 and 0.73 µm (white arrows in Figure 3 EWa and EWb, respectively) which is smaller than that detected in the cold compaction obturation technique without evidence of tag-like structures (blue arrows in Figure 3: EWa–EWc). There was no line of demarcation at the BC/gutta-percha core interface (red arrows in Figure 3: EWa–EWc). The AHP/dentin interface detected a gap ranging between 3.07 and 0.77 µm (white arrows in Figure 3 AWa–AWc), with no evidence of tag-like structures (blue arrows in Figure 3: AWa–AWc). Evidence of sealer particle diffusion within the gutta-percha core was observed without a line of demarcation between them (red arrows in Figure 3: AWc).

### 3.2. Raman Spectroscopy

**Figure 4:**
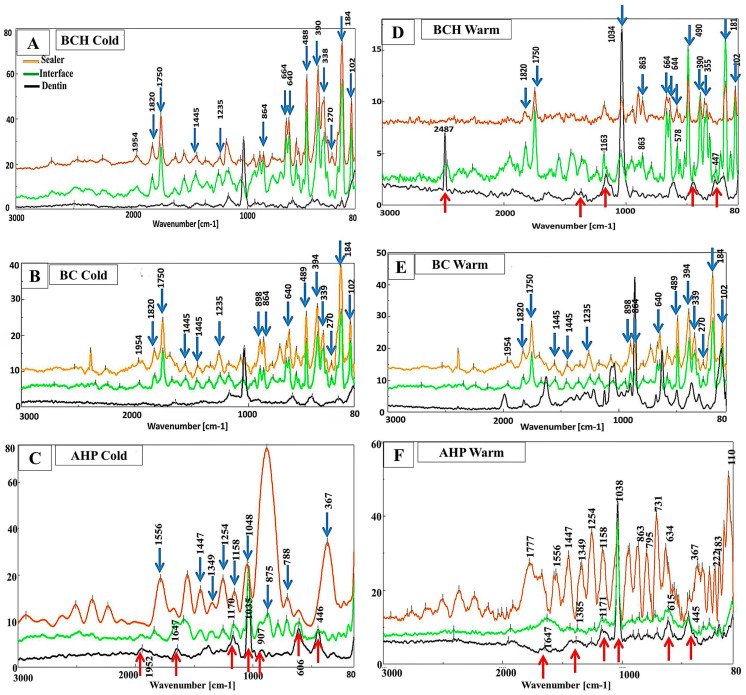
Raman spectra of the sealers, sealer/dentin interface, and adjacent dentin subjected either to cold compaction (**A**–**C**) or warm compaction (**D**–**F**). The blue arrows point to the peak of interface similar to that of the sealer, while the red arrow points to the peak of the interface similar to that of the adjacent dentin.

When bioceramic sealers (BC and BCH) were used in the cold compaction obturation technique, the spectra of the sealer/dentin interface (green lines) were identical to the spectra of the sealer (orange lines) (Figure 4A and B, respectively). Meanwhile, the spectra of the AHP/dentin interface detected sealer bands (blue arrows), and some dentin bands (red arrows) (Figure 4C).

When the sealers were subjected to heat treatment, the spectra of the BCH/dentin interface (Figure 4D) showed a mixture of sealer bands (1820, 1750, 1182, 1040, 863, 665, 645, 490, 184, and 102 cm^−1^) (blue arrows) and dentin bands (2487, 1364, 1182, 953, 447, and 263 cm^−1^) (red arrows). Meanwhile, the BC/dentin interface spectra (Figure 4E) were nearly identical to the BC sealer spectra (blue arrows) without any appearance of bands related to dentin (black line). The AHP/dentin interface spectra were nearly identical to the dentin spectra (red arrows) with no band related to the AHP sealer spectra (Figure 4F). Regarding the spectra of AHP, there was evidence of shift and intensity changes in the region of 1100–400 cm^−1^ after heat treatment (Figure 4F) compared to those subjected to the cold technique (Figure 4C). These bands were related to epoxy resin groups (1100–900 and 700–900 cm^−1^), silicate (at 510 cm^−1^), and calcium tungsten (at 840 cm^−1^) [30].

### 3.3. Confocal Laser Scanning Microscopy (CLSM)

**Figure 5:**
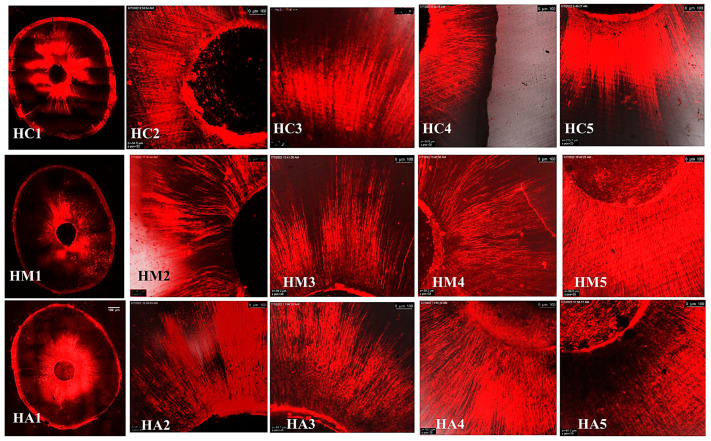
CLSM photographs (at ×2000) of root canal filled with BCH (H) at different root levels; cervical (C), middle (M), and apical (A), subjected to cold compaction obturation technique. The representing snapshot of root cross-section (1) and different views of all sides of root canal (2–5).

**Figure 6:**
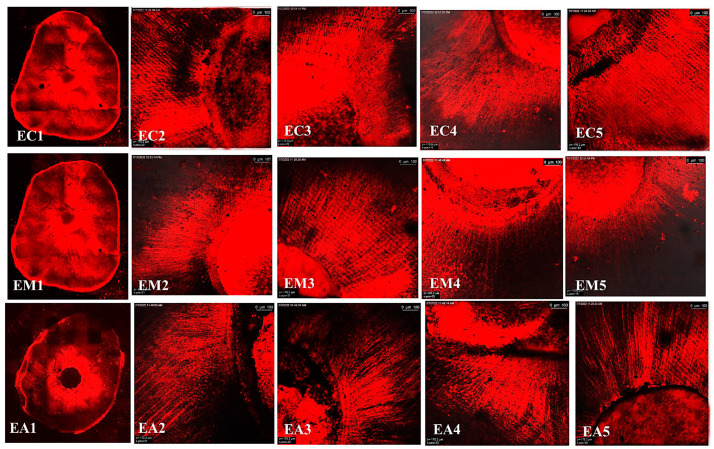
CLSM photographs (at ×2000) of root canal filled with BC (E), at different root levels; cervical (C), middle (M), and apical (A), subjected to cold compaction obturation technique. The representing snapshot of root cross-section (1) and different views of all sides of root canal (2–5).

**Figure 7:**
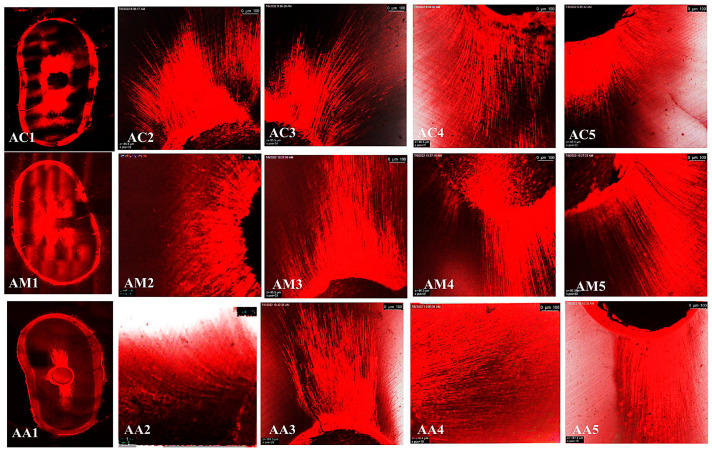
CLSM photographs (at ×2000) of root canal filled with AHP (A), at different root levels; cervical (C), middle (M), and apical (A), subjected to cold compaction obturation technique. The representing snapshot of root cross-section (1) and different views of all sides of root canal (2–5).

**Figure 8:**
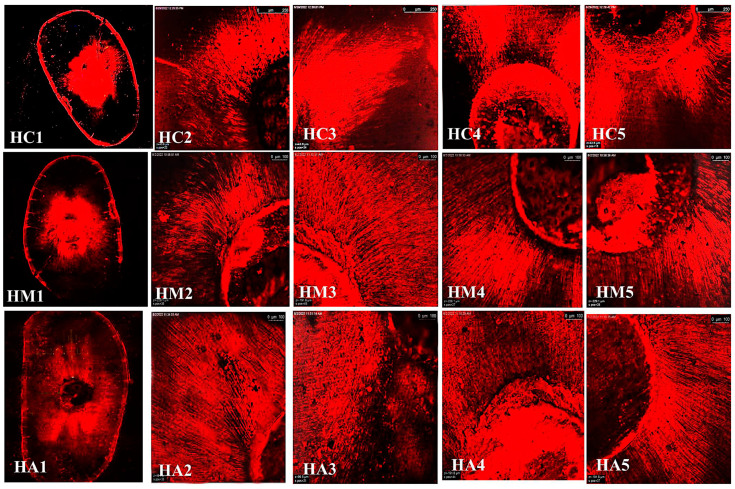
CLSM photographs (at ×2000) of root canal filled with BCH (H), at different root levels; cervical (C), middle (M), and apical (A), subjected to warm compaction obturation technique. The representing snapshot of the root cross section (1) and different views of all sides of the root canal (2–5).

**Figure 9:**
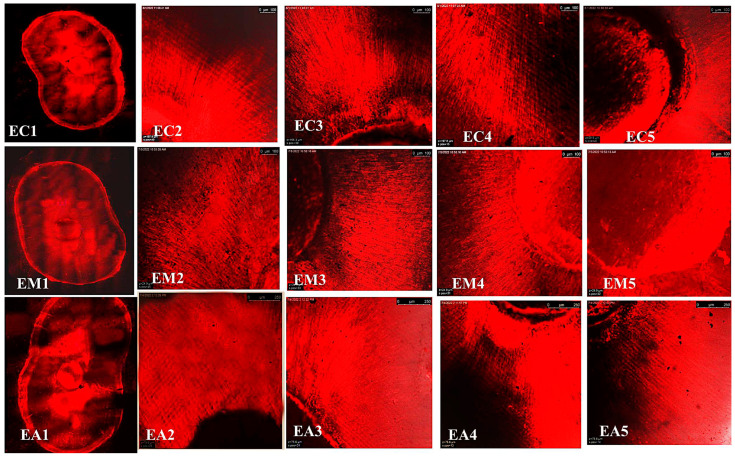
CLSM photographs (at ×2000) of root canal filled with BC (E), at different root levels; cervical (C), middle (M), and apical (A), subjected to warm compaction obturation technique. The representing snapshot of the root cross-section (1) and different views of all sides of the root canal (2–5).

**Figure 10:**
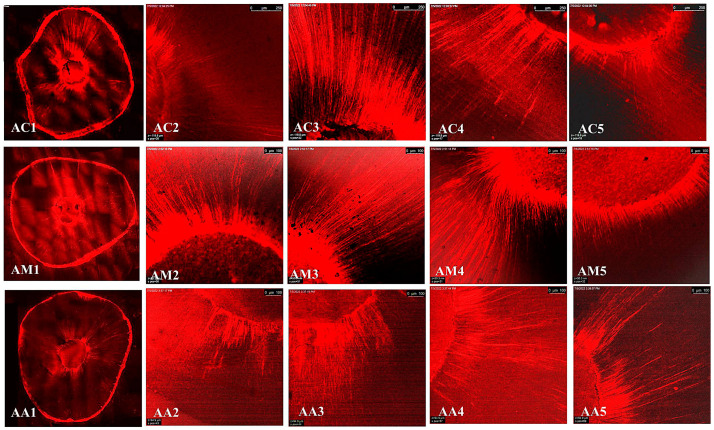
CLSM photographs (at ×2000) of root canal filled with AHP (A) at different root levels; cervical (C), middle (M), and apical (A), subjected to warm compaction obturation technique. The representing snapshot of the root cross-section (1) and different views of all sides of the root canal (2–5).

The representative images of the sealers’ distribution (BCH, BC, and AHP) associated with Rhodamine-B penetrated within the dentin after the cold and warm compaction techniques are shown in Figure 5, Figure 6 and Figure 7, Figure 8, Figure 9 and Figure 10, respectively.

#### Maximum Depth Penetration and Penetration Percent (%)

**Figure 11:**
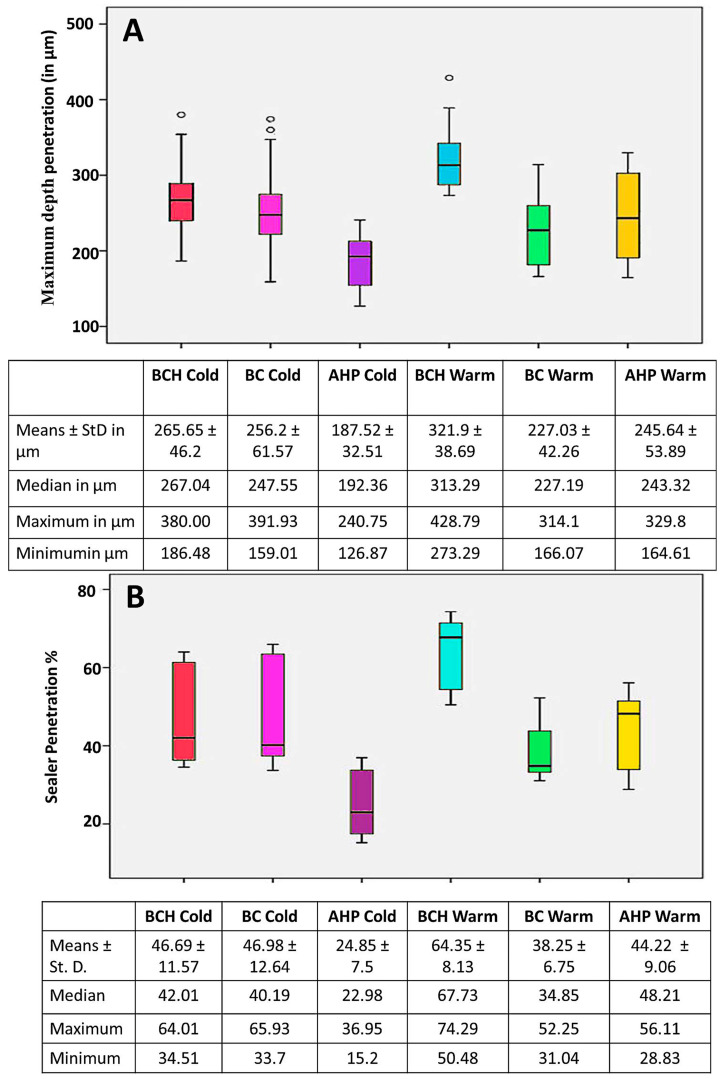
Representation of the maximum depth penetration (**A**) and penetration percent (**B**), including means ± standard deviation, median, maximum, and minimum values of all sealers subjected to either cold or warm compaction obturation techniques.

The penetration depth of the sealer was not uniform around the canal circumference. In general, the sealers exhibited greater extension in the bucco-lingual direction compared to the mesio-distal directions. Thus, the maximum depth of penetration was evaluated in the bucco-lingual direction. In the cold compaction obturation technique, AHP exhibited the least significant mean value (187.52 ± 32.51 µm at *p* < 0.001) throughout the entire length of the root canal, with no significant difference between BCH and BC sealers (265.65 ± 46.2 and 256.2 ± 61.57 µm, respectively, at *p* > 0.05). However, in the warm compaction obturation technique, BCH exhibited the significantly greatest mean value of penetration (321.9 ± 38.69 µm), followed by AHP (245.64 ± 53.89 µm), while BC exhibited the most significantly lowest mean value (227.03 ± 42.26 µm) at *p <* 0.001. The penetration percentage of each individual sealer was significantly higher in the warm compaction technique compared to the cold compaction technique. The greatest penetration percentage values were exhibited by BCH followed by AHP. BC showed the significantly lowest values (*p* < 0.001). The data obtained from all sealers (including minimum, maximum, median, and means ± standard deviation) are seen in Figure 11A for depth penetration and Figure 11B for penetration percentage.

### 3.4. Flow and Film Thickness

At a temperature of 25 °C, BC exhibited the significantly greatest mean value of flowability (23.35 ± 0.47 and median 23 mm) with no significant difference between BCH and AHP (19.45 ± 1.09 and 18.35 ± 0.47, respectively; median 20 and 18 mm, respectively). However, when the sealer was subjected to high temperature (100 °C), the flowability of both BCH and BC significantly increased (25.4 ± 1.26 and 24.35 ± 0.47 mm; median 25 and 24 mm, respectively), while AHP exhibited the significantly lowest values of flowability (19.35 ± 0.47 mm) similar to the cold compaction technique (Table 1). BCH exhibited the thinnest film thickness on the slide test upon either cold or warm compaction (0.4 µm) while BC exhibited the thickest film thickness, particularly when subjected to high temperatures (0.8 µm) (Table 1).

## 4. Discussion

Gutta-percha is known as an adhesion-free material [31]. Therefore, the root canal sealer is essential to compensate for this property and allow good sealing and intimate adaptation for root canal obturation [5,6].

Numerous root canal sealers are offered on the market to enhance the effectiveness and good adaptation of root canal obturation. A new BCH sealer was implemented in the endodontic market to fulfill the requirement of the warm compaction technique [18]. The current study compared two bioceramic sealers (BC and BCH) versus the gold standard epoxy resin AHP sealers, when BC-coated GP points were present. SEM and Raman spectroscopy were used to evaluate the sealer/dentin and sealer/gutta-percha interfaces, while CLSM determined the sealer depth penetration.

The sample size in the current study was determined according to previous studies that used three to five samples [2,24]. Because the sample size is considered small, this is “a preliminary report”.

Recently, a full obturation system including a new gutta-percha point (BC points and BC points 150 series) was introduced by Brasseler [18]. According to manufacturer’s recommendations, the BC points are advised to be used with regular BC, during the cold compaction technique. However, they undergo dehydration and cannot sustain high temperatures during the warm compaction technique [18,20]. Recently, the BC points 150 series was recommended to be used with BCH sealer, particularly during the warm compaction technique. For standardization of the current study, BC points were used with BC sealer, while BC points 150 series were used with BCH, either in the cold or warm compaction techniques.

The sealing ability and adaptation are influenced by the presence of voids/gaps either at the sealer/dentin or sealer/gutta-percha interfaces. The voids/gaps at the interface permit bacterial leakage, which is responsible for endodontic failure [32]. When the sealer was subjected to the cold compaction technique, SEM revealed gaps at the sealer/dentin interface, with a variable range according to the sealer used. The least significant gaps at the sealer/dentin interface were exhibited by AHP, followed by BCH, whereas BC showed marked gaps all over the area (ranging between 6.68 and 2.99 µm). BCH sealer showed gaps in a few areas (16.55–7.21 µm) of a greater size than those detected in BC with the cold compaction technique. This finding was also confirmed by Raman spectroscopy, which revealed that the spectra of the BC and BCH/dentin interfaces (green lines in Figure 4A,B) with the cold compaction technique were identical to those of the sealers, with no band present in the dentin spectra. It indicates the presence of gaps between the sealer and dentin and a lack of mechanical interlocking. Concerning AHP, previous studies showed a gap-free sealer/dentin interface when AHP was used with the cold compaction technique, while some porosities were observed within the sealer layer [33].

The physical and chemical properties of root canal sealers play a crucial role in their adaptation and penetration into the dentinal tubules. Bioceramic sealers, such as BC and BCH, are alkaline in nature due to the presence of calcium hydroxide byproduct, which can lead to the denaturation of dentinal collagen fibers that permit the penetration of sealer within dentinal tubules [34]. On the other hand, epoxy-resin-based sealers like AHP form a chemical covalent bond with the collagen of radicular dentin, resulting in fewer gaps at the sealer/dentin interface [35]. While bioceramic sealers have good flowability, they lack the ability to chemically or mechanically adhere to dentin. Their flow ranges between 23.1 and 26.96 mm [36]. This was confirmed in the results, as 23.35 ± 0.47 mm was recorded for BC sealer and 19.45 ± 1.09 mm for BCH sealer [34].

Furthermore, the flowability and film thickness of the sealer are important factors influencing its adaptation. Sufficient flow capacity enables the filling to spread within uneven canals and deeply penetrate dentinal tubules for the formation of monoblocks. However, excessive flow that exceeds the acceptable level, according to the international Organization for Standardization [27], can cause apical extrusion, causing harm to the surrounding tissues and periapical inflammation [16,37]. The current study revealed adequate flow for the three sealers used, within the acceptable level, with significantly greater flow in BC (*p* < 0.05) and no significant difference between BCH and AHP (*p* > 0.05). The greater flowability of BC may be attributed to its prolonged setting time [38].

Sealer film thickness is another important factor influencing adaptation. An adequate film thickness ensure satisfactory distribution of the sealer into anatomical irregularities [37]. However, a high film thickness is undesirable as it may interfere with gutta-percha adaptation to radicular dentin [37]. It may decrease the contact surface required for its adaptation to the dentin, with a subsequent increase in bacterial leakage [35]. It also increases polymerization shrinkage and solubility over time [35,39], thus inversely affecting its sealing ability. When the sealer was subjected to the cold compaction technique, both bioceramic (BCH and BC) sealers exhibited a significantly thinner film compared to AHP under the SEM. The thicker film of AHP can be attributed to its fast setting [8,40]. However, a previous study has reported different findings, with some suggesting that BC has a thicker film than AHP [35]. The discrepancy may be related to the quick solidification and lack of shrinkage of BC due to its calcium silicate content [35].

Furthermore, when the sealers were subjected to the warm compaction technique, all sealers showed a significantly thinner film, with BCH demonstrating superior results followed by AHP. This result is in accordance with a previous study [35]. The heat application during warm compaction increases the flowability of the sealer, which can prolong the setting time. Then, during compaction, pressure promotes a reduction in the sealer film thickness. The high temperature could decrease the film thickness, as determined by lab slide tests (Table 1). Conversely, other studies have found no significant effect. Chen et al., 2020, reported that high temperatures increase the film thickness of BC sealer, while not affecting BCH [41]. Yamauchi et al., 2021, determined that the heat (100 °C) did not affect the film thickness of BC sealer, while it increased BCH film thickness [28]. They quoted that “the heating may accelerate the setting reaction with increasing film thickness of calcium silicate sealers, owing to increase its volume” [28]

Flowability is another important property that affects the film thickness of the sealer. The warm compaction technique generally increases sealer flowability, resulting in a decrease in film thickness due to the compressive force applied during the compaction technique. In the current study, BCH and AHP showed a significantly smaller flow circle (19.45 ± 1.09 and 18.35 ± 0.47 mm, respectively, at room temperature) compared to BC (23.35 ± 0.47mm). The flowability of BCH further increased upon heat treatment (25.4 ± 1.26 mm at 100 °C) compared to BC and AHP (24.35 ± 0.47 and 19.35 ± 0.47 mm, respectively). Heat exhibited a further increase in BCH flowability. Similar findings have been reported previously, where BC and BCH showed a higher flow with a temperature of 100 °C [28]. However, as study by Donnermeyer et al. in 2021 reported that the flow and film thickness were not affected by elevated temperatures [19]. The flowability of sealers is influenced by their composition. Bioceramic sealers contain calcium hydroxide byproduct, which can decrease their flow rate, while AHP contains epoxy resin, which leads to a higher flow rate as reported by a previous study (37.47 mm) [42].

In general, the composition of the sealer, along with its chemical and physical properties, plays a significant role in determining the quality of the film thickness [35,41]. BC sealer contains a thickening agent which may contribute to its thick film, whereas BCH lacks a thickening agent and becomes less viscous when heated [19,41]. Furthermore, BC’s high calcium silicate content increases its film thickness and hinders sufficient polymerization shrinkage throughout its lengthy setting reaction [35] However, this is offset by BCH’s quick setting time [38].

The presence and/or absence of adaptation can be confirmed by Raman spectroscopy analysis. If the spectra of the sealer/dentin interface detected bands similar to those of the adjacent dentin, it indicates that the sealer interacts with dentin, forming a chemical interaction or mechanical interlocking; this suggests bonding of the sealer to dentin and good adhesion. However, if these bands are absent and only bands similar to those of the sealer are detected, it indicates the presence of a gap at the sealer/dentin interface.

The SEM results in the study were supported by Raman spectra analysis, as the AHP/dentin interface showed sealer bands (blue arrows) and some dentin bands (red arrows) (Figure 4C). Furthermore, the good adaptation of AHP may be attributed to its slightly acidic nature that permits self-etching to dentin, which allows for interfacial bonding and close contact with dentin [35]. In contrast, bioceramic sealers may undergo polymerization shrinkage during their setting reaction [35]. The sealer/dentin interfacial gaps exhibited non-homogenous filling with a great percentage of sealer, associated with great voids with the cold compaction technique [35,43]. However, with the warm compaction technique, superior adaptation for BCH was observed with a gap-free sealer/dentin interface at the majority of dentin walls. This was verified by the Raman spectra of the BCH/dentin interface (Figure 4D), where a mixture of sealer bands (indicated by blue arrows) and dentin bands (indicated by red arrows) were detected. Thus, an interaction between BCH sealer and adjacent radicular dentin, with good mechanical interlocking, is evident.

Although heat improved the adaptation of BC and AHP at the sealer/dentin interface, small gaps were detected in a range between 7.08 and 0.73 and 3.07 and 0.77 µm, respectively (Figure 3; Ewa–EWc and AWa–AWc. respectively). It has been suggested that the increase in temperature could reduce the viscosity and improve the flow of AHP, thereby decreasing the gaps/voids [44]. In contrast, the setting reaction of BC is accelerated at a higher temperature, which significantly reduces the flow of BC sealer. [28]. This may explain the presence of large voids in BC compared to AHP. However, BCH demonstrates compatibility with heat, which improves its flowability and adhesion properties [18].

The Raman spectra of the BC/dentin interface were identical to those of BC sealer with no bands related to dentin (Figure 4E). This indicated the presence of a gap and a lack of mechanical and chemical bonding to the dentin surface. Further, Cimpean et al. (2022) suggested that the warm compaction technique is not recommended with BC sealer, as it interferes with its adhesion properties and induces some chemical changes in the material structure [21]. On the other hand, the spectra of the AHP/dentin interface were nearly identical to the dentin spectra with no band related to those present in the AHP spectra. It appears that the heat treatment could compromise the adhesion of the resin sealer to the dentin wall. Viapiana (2014) concluded that, using system B, the temperature would rise along with AHP (+50 °C), activating the polymerization process and negatively affecting its physical properties as it would induce cross-linked polymerization and result in porosities toward the sealer layer [45]. These findings were supported by Raman spectroscopy which detected changes in band intensity and shifts in the 1100–400 cm^−1^ region. This region is associated with epoxy resin groups (1100–900 and 700–900 cm^−1^), silicate (at 510 cm^−1^), and calcium tungsten (at 840 cm^−1^) [30]. These results confirm that heat has an impact on the compositional changes in epoxy resin sealants.

It is worth mentioning that monoblock filling refers to the obturation of the root canal space with a single unit, creating a gap-free and homogeneously adapted root canal obturation [46]. The concept of a tag-like structure or monoblock is an important feature, as the sealer bonds to both the radicular dentin (from one side) and the gutta-percha core (from the other side), resulting in a single unit that resists microleakage and improves adhesion and the sealing ability of the root canal obturation, thus eliminating apical periodontitis [47,48]. Flowability is an essential property for filling canal irregularities, penetrating deep into dentinal tubules, enhancing mechanical interlocking, and creating the tag-like structure necessary for achieving the monoblock phenomenon. The ideal requirement for a root canal sealer is that the flow disc diameter should be ≥17 mm [27]. In the current study, all the investigated sealers met this criterion. The tag-like structure was detected by SEM in BCH in both the cold and warm compaction technique groups, with greater evidence observed when subjected to the warm technique. On the contrary, shallow penetration of BC sealer and no penetration of AHP sealer were detected. This finding may be attributed to BCH’s greater flowability, particularly upon heat treatment. This was confirmed by CLSM, as the greatest sealer penetration was obtained with BCH, further increasing upon heat treatment (Figure 8 and Figure 11B) The Rhodamine B dye penetration in AHP, either with the cold or warm technique (Figure 7 and Figure 10), appeared lighter compared to BCH and BC (Figure 5, Figure 6, Figure 8 and Figure 9, respectively). This supports the SEM finding, as there was a lack of tag-like structures in the AHP groups (Figure 2 ACc), while evidence of BCH sealer penetration into the dentin was greater than that of BC sealer (Figure 2 HCc and ECc). This finding confirms the excellent flowability of BCH sealer. In addition, manufacturers have introduced BC gutta-percha points (BC Points) which possess a surface impregnated with nano-bioactive bioceramic particles [49]. This structure can achieve a monoblock phenomenon, as the surface-impregnated nano-bioactive bioceramic particles of BC points chemically bond with the bioceramic-based sealer, creating a homogenous, gap-free, single-unit obturation [18,50]. This concept was confirmed by the SEM findings of the current study, as both bioceramic sealers (BC and BCH) subjected to either the cold (Figure 2 HCB and ECb, respectively) or warm (Figure 3 Hwa–HWC and Ewa–EWc, respectively) compaction technique showed nearly homogenous obturation without a line of demarcation between the sealer and gutta-percha core, while a few voids were detected within the BCH cold sealer layer. The lack of a line of demarcation at the sealer/gutta-percha interface could indicate a homogenous obturation. It has been suggested that the bioceramic system can achieve chemo-mechanical retention similar to a tertiary monoblock, as the bioceramic particles of the sealer and gutta-perch bond with each other [51]. Inconsistently, Al-Haddad (2018) reported the bonding strength of BC gutta-percha to BC sealer [5]. Unlike bioceramic sealers, AHP could not achieve the monoblock phenomenon, as SEM examination revealed a line of demarcation at the AHP/gutta-percha interface.

## 5. Limitations

Within the limitations of this study, it can be concluded that BCH, in combination with BC-impregnated gutta-percha points, can provide a gold standard 3D obturation characterized by a single-unit obturation, being gap-free and exhibiting the monoblock phenomenon.

## 6. Conclusions

The use of BC systems using either BC or BCH sealers in combination with BC points exhibited a gap-free obturation, regardless of whether a cold or warm vertical compaction technique was employed. The nano-bioactive bioceramic particles impregnanted on the surface of BC points allowed for a single-unit obturation, as there was no line of demarcation at the sealer/gutta-percha interface.

Furthermore, when the warm compaction technique was used, BCH sealer exhibited uniform and gap-free projection into the dentinal tubules, along with the formation of a tag-like structure, attaining the monoblock phenomenon that was not possible with either BC or AHP.

## Figures and Tables

**Table 1:** Representation of the means ± SD (in µm) of the film thickness of the three sealers detected by SEM images.

	Technique	Cold Compaction			Warm Compaction		
	Sealers	BCH	BC	AHP	BCH	BC	AHP
Means ± SD (in µm) of Film thickness detected by SEM images.	at cervical	25.98 ± 5.58	40.98 ± 6.29	65.39 ± 6.37	22.93 ± 4.27	24.01 ± 6.22 *	22.55 ± 2.09
	at Middle	21.57 ± 7.14	33.66 ± 5.29	64.89 ± 9.53 *	15.66 ± 5.4	21.15 ± 3.4 *	18.53 ± 2.77
	at apical	21.56 ± 4.46	26.34 ± 3.81	57.84 ± 9.75 *	11.3 ± 1.72	16.7 ± 1.54 *	10.67 ± 0.85
Flow (mm) on glass plate Means ± SD (in mm) Median		19.45 ± 1.09 (20)	23.35 ± 0.47 * (23)	18.35 ± 0.47 (18)	25.4 ± 1.26 (25.00)	24.35 ± 0.47 * (24.00)	19.35 ± 0.47 (19)
Film thickness on glass plate (µm)		0.4	0.55 *	0.5	0.4	0.8 *	0.5

Symbol (*) denotes the significantly greatest value at *p* < 0.05.

## Data Availability

All authors agree to make the results of this manuscript available for all readers, including links to publicly archived datasets analyzed or generated during the study.
